# Ubiquitin-Specific Protease 14 Negatively Regulates Toll-Like Receptor 4-Mediated Signaling and Autophagy Induction by Inhibiting Ubiquitination of TAK1-Binding Protein 2 and Beclin 1

**DOI:** 10.3389/fimmu.2017.01827

**Published:** 2017-12-15

**Authors:** Yoon Min, Sena Lee, Mi-Jeong Kim, Eunyoung Chun, Ki-Young Lee

**Affiliations:** ^1^Department of Molecular Cell Biology, Samsung Biomedical Research Institute, Sungkyunkwan University School of Medicine, Suwon, South Korea; ^2^Department of Immunology and Infectious Diseases, Harvard School of Public Health, Boston, MA, United States; ^3^The Department of Medicine, Harvard Medical School, Boston, MA, United States; ^4^Samsung Medical Center, Seoul, South Korea; ^5^Department of Health Sciences and Technology, Samsung Advanced Institute for Health Sciences & Technology, Samsung Medical Center, Sungkyunkwan University, Seoul, South Korea

**Keywords:** autophagy, Beclin 1, toll-like receptor 4, tumor necrosis factor receptor-associated factor 6, TAK1-binding protein 2, ubiquitination

## Abstract

Ubiquitin-specific protease 14 (USP14), one of three proteasome-associated deubiquitinating enzymes, has multifunctional roles in cellular context. Here, we report a novel molecular mechanism and function of USP14 in regulating autophagy induction and nuclear factor-kappa B (NF-κB) activation induced by toll-like receptor (TLR) 4 (TLR4). USP14 interacted with tumor necrosis factor (TNF) receptor-associated factor 6 (TRAF6) and interrupted the association of Beclin 1 with TRAF6, leading to inhibition of TRAF6-mediated ubiquitination of Beclin 1. Reduced expression of USP14 in USP14-knockdown (USP14^KD^) THP-1 cells enhanced autophagy induction upon TLR4 stimulation as shown by the increased conversion of cytosolic LC3-I to membrane-bound LC3-II. Moreover, USP14^KD^ human breast carcinoma MDA-MB-231 cells and USP14^KD^ human hepatic adenocarcinoma SK-HEP-1 cells showed increased cell migration and invasion, indicating that USP14 is negatively implicated in the cancer progression by the inhibition of autophagy induction. Furthermore, we found that USP14 interacted with TAK1-binding protein (TAB) 2 protein and induced deubiquitination of TAB 2, a key factor in the activation of NF-κB. Functionally, overexpression of USP14 suppressed TLR4-induced activation of NF-κB. In contrast, USP14^KD^ THP-1 cells showed enhanced activation of NF-κB, NF-κB-dependent gene expression, and production of pro-inflammatory cytokines such as IL-6, IL-1β, and tumor necrosis factor-α. Taken together, our data demonstrate that USP14 can negatively regulate autophagy induction by inhibiting Beclin 1 ubiquitination, interrupting association between TRAF6 and Beclin 1, and affecting TLR4-induced activation of NF-κB through deubiquitination of TAB 2 protein.

## Introduction

Ubiquitin-specific proteases (USPs) are deubiquitinating enzymes (DUBs) that play a special role in rescuing proteins from degradation by trimming ubiquitin chains from their substrate-distal tips (1–5). Recent evidence has shown that several USPs are critically involved in multicellular processes, including signaling of potential cellular oncogenesis and innate immunity (6, 7). Ubiquitin-specific protease 14 (USP14) as one of three proteasome-associated DUBs is a major regulator of proteasomal degradation and implicated in the development and progression of several tumors (3, 8–12). The downregulation of USP14 significantly inhibited breast cancer cell proliferation and metastasis (12). In lung adenocarcinoma, overexpression of USP14 promoted cell proliferation through the accumulation of β-catenin (9). Moreover, the high expression of USP14 was significantly correlated with tumor grade, clinical stage, and lymphatic metastasis of epithelial ovarian cancer (11). A recent report has shown that deubiquitination of disheveled (Dvl) by USP14 is required for Wnt signaling (7). USP14 regulated the ubiquitination of Dvl and its subsequent phosphorylation, which is essential for the activation of the downstream Wnt signaling (7). It has been reported that USP14 regulates autophagy by suppressing K63 ubiquitination of Beclin 1 (13). Activation of USP14 by Akt-mediated phosphorylation modulated autophagy through controlling K63 deubiquitination of Beclin 1 (13). Nevertheless, the precise molecular mechanism by which USP14 suppresses Beclin 1 remains unclear.

Autophagy and ubiquitin–proteasome system as major intracellular degradative mechanisms are regulated at both transcriptional and posttranslational level in response to different signaling pathways (14–16). Autophagy also delivers cytoplasmic constituents to autophagolysosomes to be associated with the innate immunity (17, 18). The innate immunity senses infections by recognizing essential and conserved components of pathogens known as pathogen-associated molecular patterns (PAMPs) (19). Innate immune cells such as macrophages, dendritic cells, and neutrophils express several families of pattern-recognition receptors (PRRs) that can mediate phagocytosis by recognizing different PAMPs. Toll-like receptors (TLRs), one of major PRRs, play pivotal roles in mounting the innate immunity against different pathogens through intracellular signaling pathways by inducing antimicrobial factors and inflammatory mediators (19, 20). In TLR-mediated signaling, tumor necrosis factor (TNF) receptor-associated factor 6 (TRAF6) associated with dimeric ubiquitin-conjugating enzyme Ubc13/Uev1A functions as both an adaptor and an E3 ubiquitin ligase by conjugating K63-linked ubiquitin chain to other proteins (21–23). TRAF6 ubiquitination is involved in the activation of ubiquitin-dependent kinase TAK1, after which, TAK1 can bind to several different proteins, including TAK1-binding protein (TAB) 1, TAB 2, TAB 3, and TAB 4 (20–22). TAB 2 is ubiquitinated by TRAF6, which facilitates assembly of a toll/interleukin-1 (IL-1) signaling complex containing TRAF6, TAK1, and IκB kinase (24), leading to the activation of nuclear factor-kappa B (NF-κB) and the production of pro-inflammatory cytokines (24–28). A recent study has shown that autophagy facilitates toll-like receptor (TLR) 4 (TLR4)- and TLR3-triggered migration and invasion of lung cancer cells through promoting TRAF6 ubiquitination (29). TLR4 and TLR3 activation induced autophagy *via* the TICAM1 adaptor in lung cancer cells, and that this in turn, promoted ubiquitination of TRAF6 that was essential for TLR4- and TLR3-triggered increase in the production of multiple cytokines, including IL-6, CCL2, CCL20, VEGFA, and MMP2, leading to the enhanced cell migration and invasion (29). Moreover, it has been reported that TRAF6 regulates lysine 63-linked ubiquitination of Beclin 1 to control TLR4-induced autophagy (30). TLR4 signaling induced the modification of Beclin 1 through the addition of K63-linked ubiquitin chains by TRAF6, and that contributed to the induction of autophagy, strongly supposing that TRAF6 is essential for both NF-κB activation and autophagy induction upon TLR4 stimulation.

Based on these previous findings, we hypothesized that the suppression of Beclin 1 ubiquitination by USP14 might be critically associated with TRAF6-mediated ubiquitination in both autophagy and TLR4-mediated signaling. Our data demonstrated that USP14 and Beclin 1 competitively interacted with the coiled coil (CC) domain of TRAF6 and that inhibition of Beclin 1 ubiquitination negatively affected autophagy induction. Furthermore, we demonstrated that USP14 induced deubiquitination of TAB 2, a ubiquitination substrate of TRAF6, thereby suppressing the activation of TLR4-mediated signaling molecules such as TAK1 and IKKs, leading to inhibition of NF-κB activation upon TLR4 stimulation. Taken together, our data provide a novel regulatory mechanism of USP14 in autophagy induction and activation of NF-κB induced by TLR4.

## Materials and Methods

### Cell Lines and Reagents

HEK293T human embryonic kidney cells were purchased from the American Type Culture Collection (ATCC, Manassas, VA, USA) and maintained in Dulbecco’s modified Eagle’s medium (DMEM) (Invitrogen, Carlsbad, CA, USA). HEK293 cells expressing human TLR4 (293/TLR4) were purchased from InvivoGen (San Diego, CA, USA) and maintained in DMEM containing 4.5 g/l glucose, 2–4 mM l-glutamine, 10% fetal bovine serum (FBS), 50 U/ml penicillin, 50 µg/ml streptomycin, 100 µg/ml Normocin according to the manufacturer’s protocol. THP-1 human monocytic cells were purchased from ATCC and maintained in RPMI medium (Invitrogen) containing 10% FBS, 2 mM l-glutamine, 100 U/ml penicillin, 100 µg/ml streptomycin, and 5 × 10^−5^ M β-mercaptoethanol. Human breast carcinoma cell line MDA-MB-231 and human hepatic adenocarcinoma cell line SK-HEP-1 were obtained from ATCC and maintained in DMEM (Invitrogen) supplemented with 10% FBS. Dimethyl sulfoxide (DMSO, D8418), 3-methyladenine (3-MA, M9281), and pepstatin A (P4265) were purchased from Sigma (Sigma-Aldrich, St. Louis, MO, USA). Stock solutions were prepared in DMSO. The final concentration of DMSO in culture medium was <0.2% volume.

### Generation of USP14-Knockdown Cells

Lentivirus containing small hairpin RNA (shRNA) targeting human USP14 (sc-76817-V) and control shRNA lentivirus (sc-108080) were purchased from Santa Cruz Biotechnology (Santa Cruz, CA, USA). Cells were cultured in 24-well cell culture plate at density of 2 × 10^4^ cells per well and infected with control shRNA lentivirus or USP14 shRNA lentivirus according to the manufacturer’s protocol. Control (Ctrl) THP-1 cells, USP14-knockdown (USP14^KD^) THP-1 cells, Ctrl MDA-MB-231 cells, USP14^KD^ MDA-MB-231 cells, Ctrl SK-HEP-1 cells, and USP14^KD^ SK-HEP-1 cells were cultured in puromycin-containing (4–8 µg/ml) medium for 2 weeks to select stable clones and cultured as described previously (25, 26).

### Luciferase Reporter Assay

Luciferase reporter assay was performed as described previously (25, 26, 28). Briefly, 293/TLR4 cells were transiently transfected with mock as control vector or Flag-USP14 vector by using Lipofectamine 2000 (Invitrogen). After 24 h of culturing, cells were transiently transfected with NF-κB-dependent reporter construct pBIIx-luc and Renilla luciferase vector (Promega, Madison, WI, USA). Cells were treated with or without 200 ng/ml lipopolysaccharide (LPS; L6143, Sigma-Aldrich) for 6 h and luciferase activity was measured using dual-luciferase assay kit (Promega).

### Measurement of Proinflammatory Cytokines and p65 or p50 DNA-Binding Assay

Ctrl or USP14^KD^ THP-1 cells were treated with or without LPS (200 ng/ml) for 9 h and culture supernatants were harvested. Levels of human TNF-α, IL-1β, and IL-6 were measured in these supernatants using respective enzyme-linked immunosorbent assay (ELISA) kits (R&D Systems, Minneapolis, MN, USA) per the manufacturer’s protocol (31, 32). For p65 or p50 DNA-binding assay, control (Ctrl) or USP14^KD^ THP-1 cells were treated with or without LPS (200 ng/ml) for 6 h. Nuclear proteins were then prepared with CelLytic NuCLEAR extraction kit (Sigma-Aldrich) per the manufacturer’s protocol. Activities of transcription factors p65 or p50 were determined using TransAM NF-κB transcription factor assay kit (Active Motif North America, Carlsbad, CA, USA) per the manufacturer’s instructions (25, 26).

### Intracellular Cytokine Assay

Intracellular cytokine production was detected in Ctrl or USP14^KD^ THP-1 cells treated with or without LPS (200 ng/ml) for 12 h. Brefeldin A (10 µg/ml, Sigma-Aldrich) was added at 4 h before completion of incubation with stimuli. Cells were permeabilized using FACS permeabilizing solution (Becton Dickinson, Franklin Lakes, NJ, USA) for 10 min at room temperature. After washing with FACS buffer (PBS with 0.5% bovine serum albumin and 0.1% NaN_3_), cells were incubated at room temperature for 30 min with the following monoclonal antibodies: FITC-conjugated anti-human interleukin 1 beta (IL-1β), IL-6, TNF alpha (TNF-α) purchased from Becton Dickinson (San Jose, CA, USA), and IgG subclass-matched control antibody obtained from BD Pharmingen (San Diego, CA, USA). Cells were washed, resuspended in 1% paraformaldehyde, and analyzed in a FACS Calibur flow cytometer (Becton Dickinson) by using Cellquest (version 3.1) software.

### Quantitative Real-time Polymerase Chain Reaction (qRT-PCR)

Ctrl and USP14^KD^ THP-1 cells were treated for different time periods with or without LPS (200 ng/ml). Total RNA was isolated and cDNA was synthesized per the manufacturer’s protocol (Qiagen, Valencia, CA, USA). Primers, hIL-6, hTNF-α, and hIL-1β were purchased from Qiagen. qRT-PCR was performed using Rotor-Gene Q (Qiagen) per the manufacturer’s protocol.

### Plasmids

Myc-tagged USP14, Myc-tagged Beclin 1, Flag-tagged USP14, Flag-tagged TRAF6, HA-tagged TAB 2, HA-tagged Ub, and Myc-tagged Ub vectors were used in this study. Myc-tagged Beclin 1 truncated mutants, Myc-tagged Beclin 1 1-269, and Myc-tagged Beclin 1 1-127 were generated using specific primers shown in Supplementary Material (Table S1 in Supplementary Material). HA-tagged TAB 2 truncated mutants, HA-tagged TAB 2 ΔCUE, HA-tagged TAB 2 1-518, and HA-tagged TAB 2 518-693 were generated with specific primers shown in Supplementary Material (Table S2 in Supplementary Material). Flag-tagged TRAF6 truncated mutants, Flag-tagged TRAF6 110-522, Flag-tagged TRAF6 260-522, and Flag-tagged TRAF6 349-522 were generated as described previously (33). Flag-tagged Beclin 1 vector was purchased from Addgene (Cambridge, MA, USA). Myc-tagged Beclin 1 vector was generated by PCR using Flag-tagged Beclin 1 as a template and inserted into pCMV-Myc (Addgene). pCDNA3-USP14 vector was kindly provided by Dr. M. J. Lee (Seoul National University College of Medicine, Seoul, Korea). Myc-tagged USP14 or Flag-tagged USP14 vector was generated by PCR using pCDNA3-USP14 as a template.

### Western Blotting and Immunoprecipitation (IP) Assays

Western blotting and IP assays were performed as described previously (25, 26, 28). Briefly, HEK293T cells were transiently transfected with mock as control vector, Flag-USP14, Myc-Beclin 1 wild type (wt), Myc-Beclin 1 truncated mutants, Flag-TRAF6 wt, Flag-TRAF6 truncated mutants, Myc-USP14 wt, HA-Ub, HA-TAB 2 wt, HA-TAB 2 truncated mutants, or Myc-Ub vector indicated in each experiment by using Lipofectamine 2000 (Invitrogen). At 38 h after transfection, transfected cells were extracted and immunoprecipitated with anti-Flag (Cell Signaling Technology, Beverly, MA, USA), anti-HA (Cell Signaling Technology), or anti-Myc antibody (Cell Signaling Technology). Immunoprecipitated complexes were separated by 6–10% sodium dodecyl sulfate polyacrylamide gel electrophoresis (SDS-PAGE) and probed with anti-HA, anti-Myc, or anti-Flag antibody. Ctrl and USP14^KD^ THP-1 cells treated with or without 3-MA (5 mM) and pepstatin A (10 µM) were stimulated with LPS (200 ng/ml) for 6 h. Whole cell lysates were subjected for immunoblot analysis of LC3A/B (4108, Cell Signaling Technology) and GAPDH (Cell Signaling Technology) as a loading control. Ctrl and USP14^KD^ THP-1 cells were treated with or without LPS (200 ng/ml) for different times. Cells were extracted, separated by 6–10% SDS-PAGE, and probed with the following antibodies: IκB-α, pho-p38, p38, pho-IKKαβ, IKKβ, and GAPDH purchased from Cell Signaling Technology (Beverley, MA, USA).

### Immunofluorescence Microscopy

Cells were grown on glass coverslips overnight, fixed with 4% paraformaldehyde (Sigma-Aldrich, P-6148), and treated with 0.2% Triton X-100 to permeabilize for 30 min on ice. To detect LC3 puncta, immunofluorescence microscopy assay was performed with anti-LC3A/B antibody (4108, Cell Signaling Technology) as described previously (24). Slides were mounted in VECTA SHIELD mounting medium (H-1000, Vector Laboratories, Burlingame, CA, USA) and examined under a LSM 710 laser-scanning confocal microscope (Carl Zeiss, Jena, Germany).

### Wound-Healing and Transwell Migration Assay

5 × 10^5^ cells were seeded into 12-well plates and grown to confluence. Cell monolayer was gently scratched with a sterile yellow Gilson-pipette tip to form a wide gap of approximately 400 µm. Cells were then rinsed with culture medium to remove floating cells and debris. Cells were treated with vehicle (DMSO, <0.2% in culture medium) or 3-MA (5 mM), and images were captured after 0, 12, or 24 h. For migration assay, transwell inserts (Costar, Corning Inc., Cat No. 3422) were placed into wells. The cells (1 × 10^5^) were suspended in DMEM containing vehicle or 3-MA (5 mM) and added to the top chambers of the transwells in 24-well plates, and DMEM with 10% FBS was added to the bottom chambers. After an overnight incubation, the cells that remained in the top chamber (non-migrated) were removed, and the cells in the bottom chamber (migrated) were fixed and stained with crystal violet to visualize the nuclei. All experiments were conducted in triplicate and repeated twice.

### Statistical Analysis

*In vitro* data are presented as mean ± SEM from triplicate samples. Comparisons were statistically tested using Student’s *t*-test. *P*-values <0.05 or <0.01 were considered to be statistically significant.

## Results

### USP14 Competes with TRAF6 for the Interaction with Beclin 1, Thereby, Inhibiting TRAF6-Mediated Ubiquitination of Beclin 1

**Figure 1:**
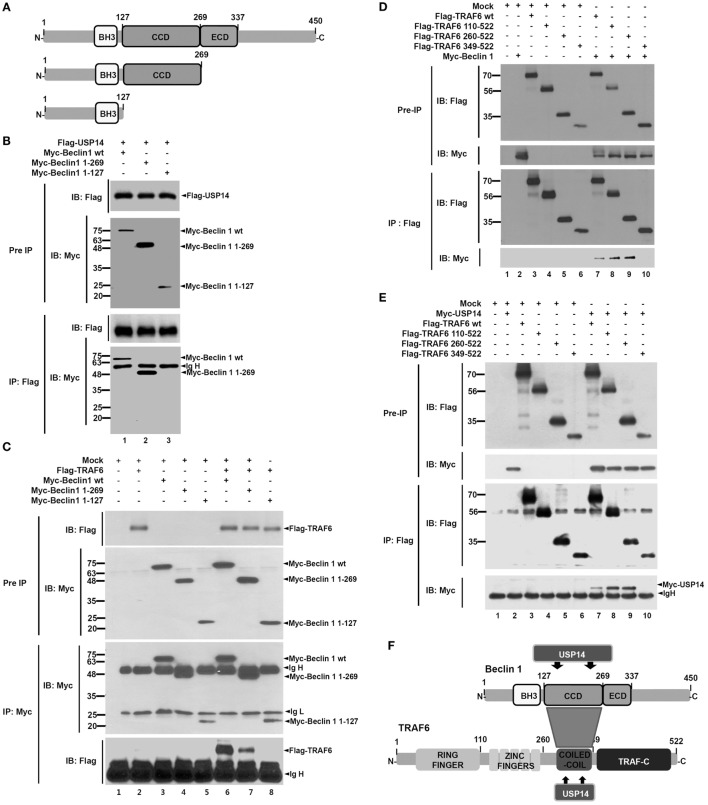
Molecular interactions among tumor necrosis factor (TNF) receptor-associated factor 6 (TRAF6), Beclin 1, and ubiquitin-specific protease 14 (USP14). **(A)** Truncated mutants of Beclin 1. Myc-tagged Beclin 1 truncated mutants were generated as described in Section “Materials and Methods.” BH3, Bcl-2 Homology 3; CCD, coiled coil domain; ECD, evolutionarily conserved domain. **(B)** Expression vectors of Myc-tagged Beclin 1 wild type (wt) and truncated mutants were co-transfected with Flag-tagged USP14 plasmid into HEK293T cells followed by immunoprecipitation (IP) and western blotting analyses. **(C)** Myc-tagged Beclin 1 wt, Myc-tagged Beclin 1 truncated mutants, and mock as control plasmid were co-transfected with Flag-tagged TRAF6 into HEK293T cells followed by IP and western blotting analyses. **(D)** Flag-tagged TRAF6 wt, Flag-tagged TRAF6 truncated mutants, and mock as control plasmid were co-transfected with Myc-tagged Beclin 1 into HEK293T cells followed by IP and western blotting analyses. **(E)** Flag-tagged TRAF6 wt, Flag-tagged TRAF6 truncated mutants, and mock as control plasmid were co-transfected with Myc-tagged USP14 into HEK293T cells followed by IP and western blotting analyses. **(F)** A schematic model showing molecular interactions among TRAF6, Beclin 1, and USP14. TRAF-C, C-terminal TRAF domain.

Ubiquitin-specific protease 14 was negatively implicated in autophagy induction by suppressing ubiquitination of Beclin 1 (13). However, the precise molecular mechanism remains unclear. A recent study has shown that TRAF6-mediated ubiquitination of Beclin 1 is critical for TLR4-triggered autophagy in macrophages (30). Therefore, we hypothesized that USP14 might be implicated in autophagy induction by regulating TRAF6-mediated ubiquitination of Beclin 1. To test this hypothesis, we first examined molecular associations among USP14, Beclin 1, and TRAF6 proteins. Co-transfection assay with Myc-Beclin 1 and Flag-USP14 in HEK293T cells revealed that Flag-USP14 interacted with Myc-Beclin 1 (Figure S1A in Supplementary Material). To characterize interaction between USP14 and Beclin 1, we generated truncated mutants of Beclin 1 (Figure 1A). Flag-USP14, Myc-Beclin 1 wild type (wt) and Myc-Beclin 1 truncated mutants were transiently transfected into HEK293T cells, and then IP assay was performed with anti-Flag antibody. Binding of USP14 with Beclin 1 was significantly abolished upon deletion of its CC domain (Figure 1B, lane 3), suggesting that the CC domain of Beclin 1 was important for the interaction (Figure S1B in Supplementary Material). We next examined interactions between TRAF6 and Beclin 1. Expression vectors of truncated Beclin 1 mutants or truncated TRAF6 mutants were co-expressed with TRAF6 or Beclin 1 in HEK293T cells and the interaction site between TRAF6 and Beclin 1 was analyzed by co-immunoprecipitation. Binding of TRAF6 with Beclin 1 was significantly reduced upon deletion of its CC domain (Figure 1C, lane 8), indicating that TRAF6 interacted with the CC domain of Beclin 1. Furthermore, the binding of Beclin 1 with TRAF6 appeared very strong through the CC domain of TRAF6 (Figure 1D, lanes 7–9). Finally, we determined domains of TRAF6 that interacted with USP14. Expression vectors of Flag-TRAF6 wt, Flag-TRAF6 truncated mutants, and Myc-USP14 were transiently transfected into HEK293T cells, and then IP assay was performed with anti-Flag antibody. As shown in Figure 1E, Flag-TRAF6 wt, Flag-TRAF6 110-522, and Flag-TRAF6 260-522, but not Flag-TRAF6 349-522, were co-precipitated with Myc-USP14, indicating that USP14 interacted with the CC domain of TRAF6. These results demonstrate that USP14 and Beclin 1 interact with the CC domain of TRAF6 while TRAF6 and USP14 interacted with the CC domain of Beclin 1 as depicted in Figure 1F.

**Figure 2:**
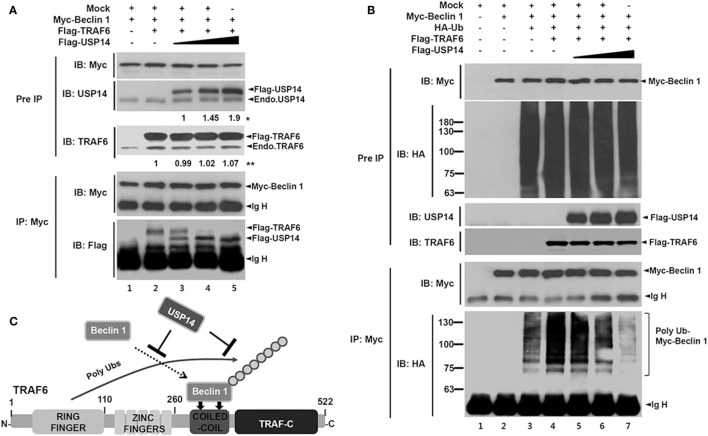
Ubiquitin-specific protease 14 (USP14) inhibits ubiquitination of Beclin 1. **(A)** Myc-tagged Beclin 1, Flag-tagged tumor necrosis factor (TNF) receptor-associated factor 6 (TRAF6), and mock as control plasmid were co-transfected with Flag-tagged USP14 plasmid at different concentrations. At 38 h post-transfection, transfected cells were extracted, immunoprecipitated with anti-Myc antibody, and subjected to immuno-blotting (IB) assay using anti-USP14, anti-TRAF6, anti-Flag, or anti-Myc antibody. *The intensity of Flag-USP14 was measured by ImageJ. Fold change relative to Flag-USP14 of lane 3 was calculated. **The intensity of Flag-TRAF6 was measured by ImageJ. Fold change relative to Flag-TRAF6 of lane 2 was calculated. **(B)** Myc-tagged Beclin 1, HA-tagged Ub, Flag-tagged TRAF6, and mock as control plasmid were co-transfected with Flag-tagged USP14 vector at different concentrations. At 38 h post-transfection, transfected cells were extracted, immunoprecipitated with anti-Myc antibody, and subjected to IB assay using anti-HA, anti-Myc, anti-USP14, or anti-TRAF6 antibody. **(C)** A schematic model showing the inhibition of Beclin 1 ubiquitination by USP14.

Since TRAF6 interacts with Beclin 1 and TRAF6-mediated ubiquitination of Beclin 1 is critical for autophagy induction (30), USP14 and Beclin 1 might competitively interact with the CC domain of TRAF6 and affect TRAF6-mediated ubiquitination of Beclin 1. To examine this possibility, expression vectors of Myc-Beclin 1 and Flag-TRAF6 were co-expressed in HEK293T cells along with different concentrations of Flag-USP14 and the interaction of TRAF6 with Beclin 1 was analyzed by co-IP. As expected, TRAF6-Beclin 1 interaction was confirmed in the absence of USP14 (Figure 2A, lane 2). Such interaction was gradually attenuated in the presence of USP14 in a dose-dependent manner (Figure 2A, lane 2 vs. lanes 3–5 in Flag-TRAF6). With increasing amount of USP14, in contrast, the interaction between Beclin 1 and USP14 was gradually increased (Figure 2A, lanes 3–5 in Flag-USP14). Although the role of USP14 cannot be completely ruled out as a proteasome-associated DUB enzyme that can affect the expression of TRAF6 (Figure 2A, IB: TRAF6), these results suggest that USP14 inhibits the interaction of Beclin 1 to TRAF6 through the competitive interaction to TRAF6 with Beclin 1. Based on this result, we further examined whether TRAF6 and USP14 interaction might affect ubiquitination of Beclin 1. Expression vectors of Myc-Beclin 1, HA-Ub, and Flag-TRAF6 were co-expressed in HEK293T cells along with different concentrations of Flag-USP14 and ubiquitination of Beclin 1 was analyzed by co-IP. Ubiquitination of Beclin 1 was markedly enhanced in the presence of Flag-TRAF6 (Figure 2B, lane 3 vs. lane 4). However, it was gradually attenuated by Flag-USP14 in a dose-dependent manner (Figure 2B, lane 4 vs. lanes 5–7). As depicted in Figure 2C, these results suggest that USP14 and Beclin 1 can competitively interact with the CC domain of TRAF6 and inhibit TRAF6-mediated ubiquitination of Beclin 1.

### USP14 Negatively Regulates Autophagy Induction

**Figure 3:**
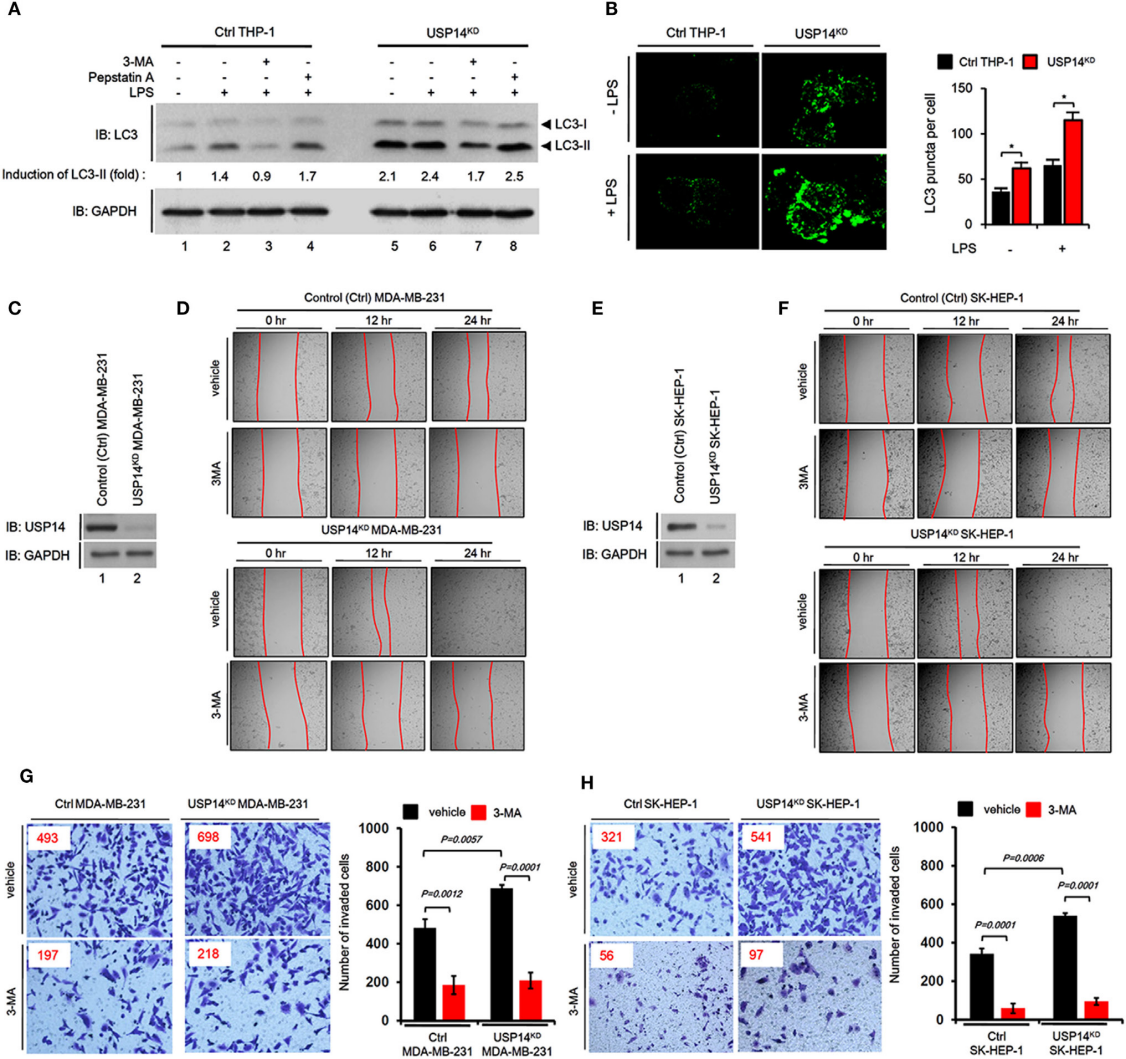
Reduction of ubiquitin-specific protease 14 (USP14) enhances autophagy induction and the ability of cancer migration and invasion in USP14-knockdown cells. **(A)** Ctrl and USP14^KD^ THP-1 cells treated with or without 3-MA (5 mM) and pepstatin A (10 µM) were stimulated with LPS (200 ng/ml) for 6 h. Whole cell lysates were subjected to immunoblot analysis of LC3A/B and GAPDH as a loading control. Band intensity was quantified using Image J software. **(B)** Ctrl and USP14^KD^ THP-1 cells were treated with or without LPS (100 ng/ml) for 16 h and then fixed. Digital images were captured with confocal microscopy (left) and the number of puncta was scored (right). Quantification shown above represents the mean ± SEM of puncta per cell (*n* = 5) from three independent experiments. *P < 0.05 **(C)** Ctrl and USP14^KD^ MDA-MB-231 cells were generated as described in Section “Materials and Methods.” Reduction of endogenous USP14 protein level was evaluated by western blotting with antibodies against USP14 and GAPDH (loading control). **(D)** Ctrl and USP14^KD^ MDA-MB-231 cells were seeded into 12-well cell culture plates, grown to confluence, and scratched with a sterile yellow Gilson-pipette tip. Cells were rinsed with culture medium to remove floating cells and debris, and then treated with vehicle (DMSO, <0.2% in culture medium) or 3-MA (5 mM), and images were captured after 0, 12, or 24 h. **(E)** Ctrl and USP14^KD^ SK-HEP-1 cells were generated as described in Section “Materials and Methods.” Reduction of endogenous USP14 protein level was evaluated by western blotting using antibodies to USP14 and GAPDH (loading control). **(F)** Ctrl and USP14^KD^ SK-HEP-1 cells were seeded into 12-well cell culture plates, grown to confluence, and scratched with a sterile yellow Gilson-pipette tip. Cells were rinsed with culture medium to remove floating cells and debris, and then treated with vehicle (DMSO, <0.2% in culture medium) or 3-MA (5 mM), and images were captured after 0, 12, or 24 h. **(G,H)** Transwell chamber was used for cell migration assay. Ctrl MDA-MB-231 cells and USP14^KD^ MDA-MB-231 cells **(G)** or Ctrl SK-HEP-1 cells and USP14^KD^ SK-HEP-1 cells **(H)** were suspended in Dulbecco’s modified Eagle’s medium containing vehicle or 3-MA (5 mM) and added to the top chambers of the transwells in 24-well plates. After an overnight incubation, cells were fixed and stained with crystal violet. Number of cell migration was counted. Results are presented as mean ± SEM of three independent experiments.

Toll-like receptor 4 signaling triggers ubiquitination of Beclin 1 through a mechanism that depends on TRAF6 and is essential for autophagy induction (30). We explored the functional role of USP14 in autophagy induction induced by TLR4 stimulation. In order to do that, we generated USP14-knockdown monocyte/macrophage THP-1 cells along with control (ctrl) THP-1 cells (Figure S2 in Supplementary Material) and examined autophagy induction upon TLR4 stimulation. Reduction of USP14 expression in USP14^KD^ THP-1 cells resulted in upregulation of LC3-II protein levels compared to those in Ctrl THP-1 cells (Figure 3A, lane 1 in Ctrl vs. lane 5 in USP14^KD^). Upon LPS stimulation, the significant increase of LC3-II protein levels could be seen in Ctrl THP-1 cells (Figure 3A, lane 1 vs. lane 2), whereas the less increased level of LC3-II proteins could be seen in USP14^KD^ THP-1 cells (Figure 3A, lane 5 vs. lane 6). These results suppose that USP14^KD^ THP-1 cells seem to have the relatively low responsiveness to LPS stimulation in the autophagy induction, which might be due to the increased basal level of LC3-II proteins as compared with that of Ctrl THP-1 cells. In contrast, co-treatment of a autophagy inhibitor 3-MA, an inhibitor of phosphatidylinositol 3-kinases, led to marked decrease of LC3-II protein levels in both cells treated LPS (Figure 3A, lane 3 in Ctrl and lane 7 in USP14^KD^), whereas significant accumulation of LC3-II protein levels could be detected in treatment of the lysosomal protease inhibitor pepstatin A (Figure 3A, lane 4 in Ctrl and lane 8 in USP14^KD^). Upon TLR4 stimulation, in addition, the number of LC3 puncta in USP14^KD^ THP-1 cells was significantly higher than that in Ctrl THP-1 cells (Figure 3B, Ctrl vs. USP14^KD^), strongly suggesting that reduced USP14 expression could promote autophagic flux upon TLR4 stimulation.

Next, we verified the biological function of USP14 in cancer progression related to autophagy. Numerous reports have shown that autophagy is critically involved in cancer progression, including cancer migration and invasion (34–37). We established stable USP14 knockdown cancer cells, USP14^KD^ human breast carcinoma MDA-MB-231 (USP14^KD^ MDA-MB-231) (Figure 3C), and human hepatic adenocarcinoma SK-HEP-1 cells (USP14^KD^ SK-HEP-1) (Figure 3E). We examined whether USP14-induced inhibition of autophagy induction was associated with capacity of cancer migration and invasion. Wound healing and transwell invasion assay were used to assess cancer migration and invasion, respectively. As shown in Figures 3D,F, the migration of USP14^KD^ MDA-MB-231 and USP14^KD^ SK-HEP-1 cells treated with vehicle was increased in a time-dependent manner (Figure 3D, USP14^KD^ MDA-MB-231 treated with vehicle vs. Ctrl MDA-MB-231 treated with vehicle; Figure 3F, USP14^KD^ SK-HEP-1 treated with vehicle vs. Ctrl SK-HEP-1 treated with vehicle). As expected, the treatment of 3-MA, a autophagy inhibitor, induced the marked inhibition of migration in both cells (Figures 3D,F, vehicle vs. 3-MA). Further study by transwell invasion assay revealed significant increases of cellular invasive ability in USP14^KD^ MDA-MB-231 and USP14^KD^ SK-HEP-1 cells compared to Ctrl MDA-MB-231 and Ctrl SK-HEP-1 cells (Figures 3G,H, Ctrl cells treated with vehicle vs. USP14^KD^ cells treated with vehicle), whereas the marked attenuation could be detected in the treatment of 3-MA (Figures 3G,H, vehicle vs. 3-MA). Taken together, these results suggest that USP14 and Beclin 1 can competitively interact with the CC domain of TRAF6 and inhibit TRAF6-mediated ubiquitination of Beclin 1, leading to decreased autophagy induction as depicted in Figure S3 in Supplementary Material.

### USP14 Negatively Regulates TLR4-Induced Activation of NF-κB

**Figure 4:**
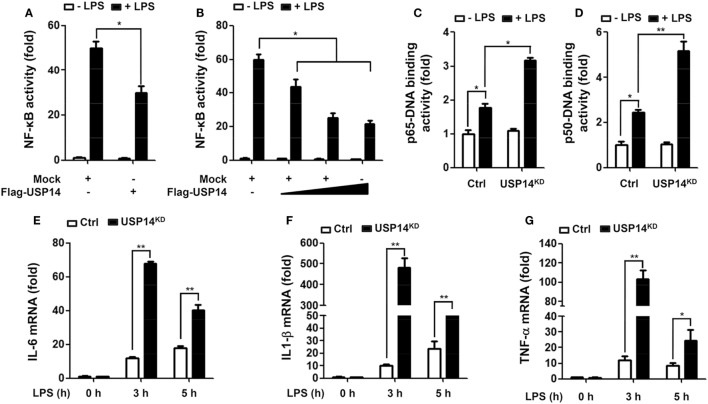
Ubiquitin-specific protease 14 (USP14) inhibits nuclear factor-kappa B activation induced by toll-like receptor 4 (TLR4). **(A)** 293/TLR4 cells were transfected with mock as control vector or Flag-tagged USP14 vector and then further transfected with with pBIIx-luc and Renilla luciferase vector, as described in Section “Materials and Methods.” Cells were treated with or without LPS (200 ng/ml) for 6 h followed by luciferase activity assay. Results are expressed as fold-induction in luciferase activity relative to that in untreated cells. All luciferase assays were repeated at least three times. All error bars represent mean ± SEM from one representative experiment of triplicate samples. **(B)** 293/TLR4 cells were transfected with mock or different concentrations of Flag-tagged USP14 vector. Luciferase reporter assay was then performed as described in **(A)**. Results are expressed as fold-induction in luciferase activity relative to that in untreated cells. All luciferase assays were repeated at least three times. All error bars represent mean ± SEM from one representative experiment of triplicate samples. **(C,D)** Ctrl and USP14^KD^ THP-1 cells were treated with or without LPS (200 ng/ml) for 6 h and then analyzed for p65-DNA binding activity **(C)** or p50-DNA binding activity. **(D)** All binding assays were repeated at least three times. All error bars represent mean ± SEM from one representative experiment of triplicate samples. **(E–G)** Ctrl and USP14^KD^ THP-1 cells were treated with or without LPS (200 ng/ml) for different time periods as indicated and analyzed by quantitative real-time polymerase chain reaction (qRT-PCR) using specific primers to IL-6 **(E)**, IL-1β **(F)**, and tumor necrosis factor (TNF)-α **(G)**. All qRT-PCR assays were repeated at least three times. All error bars represent mean ± SEM from one representative experiment of triplicate samples. *P < 0.05 and **P < 0.01.

Beside the functional role of TRAF6 ubiquitin-ligase activity in autophagy induction, TRAF6 also plays a pivotal role in the activation of NF-κB upon TLR4 stimulation (20, 21, 24–26). Deubiquitination is an important negative regulatory mechanism that reduces levels of protein ubiquitination (38, 39). Although the mechanism by which USP14 regulates K63 ubiquitination in control of cellular processes and its functional significance are not well characterized, it has been reported that the activity of USP14 in deubiquitinating K63 ubiquitin linkages is likely to be physiologically relevant (40). We, therefore, determined whether USP14 was implicated in the TLR4-mediated NF-κB activation. Overexpression of USP14 in 293/TLR4 cells attenuated NF-κB reporter activity induced by LPS stimulation (Figure 4A, mock vs. Flag-USP14) and the inhibition was dependent on the quantity of USP14 transfected (Figure 4B). To verify such results, Ctrl or USP14^KD^ THP-1 cells were treated with or without LPS and then NF-κB activation was assessed by using p65- or p50-DNA binding assay. DNA-binding activities of p65 and p50 were increased in Ctrl THP-1 cells in the presence of LPS (Figures 4C,D, open bar vs. closed bar in Ctrl) and marked enhancement was detected in USP14^KD^ THP-1 cells (Figures 4C,D, Ctrl vs. USP14^KD^).

**Figure 5:**
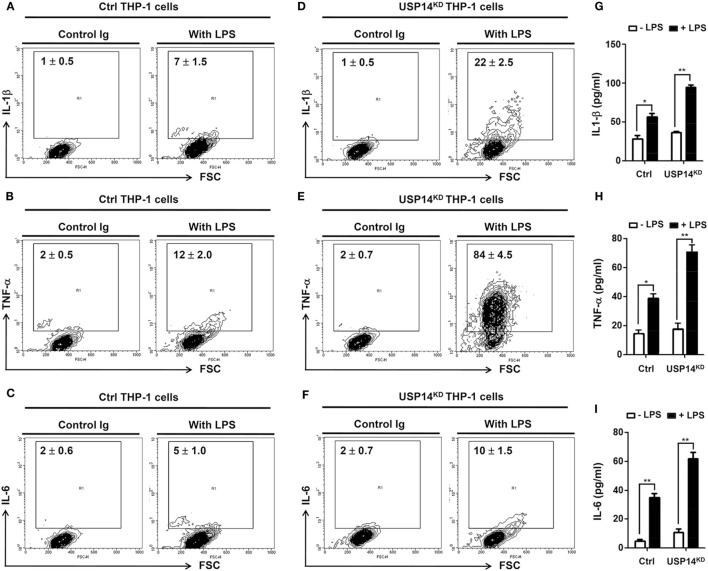
Ubiquitin-specific protease 14 knockdown in THP-1 cells enhances production of pro-inflammatory cytokines. **(A–F)** Control (Ctrl) or USP14^KD^ THP-1 cells were treated with or without LPS (200 ng/ml) for 12 h. Cells were treated with brefeldin A and permeabilized as described in Section “Materials and Methods.” After washing with FACS buffer, cells were incubated at room temperature for 30 min with the following monoclonal antibodies: FITC-conjugated anti-human interleukin 1 beta (IL-1β) **(A,D)**, FITC-conjugated anti-human tumor necrosis factor α (TNF-α) **(B,E)**, FITC-conjugated anti-human interleukin 6 (IL-6) **(C,F)**, and IgG subclass-matched control antibody. Cells were washed, resuspended in 1% paraformaldehyde, and analyzed by flow cytometry. All assays were repeated at least three times. All error bars represent mean ± SEM from one representative experiment of triplicate samples. **(G–I)** Ctrl and USP14^KD^ THP-1 cells were treated with or without LPS (200 ng/ml) for 9 h and levels of hIL-1β **(G)**, hTNF-α **(H)**, and hIL-6 **(I)** were measured by enzyme-linked immunosorbent assay (ELISA). All ELISA assays were repeated at least three times. All error bars represent mean ± SEM from one representative experiment of triplicate samples. *P < 0.05 and **P < 0.01.

It is known that TLR4 stimulation induces the activation of NF-κB, leading to the expression of pro-inflammatory cytokines (20, 21, 24–26). We further examined whether reduction of USP14 affected the expression of pro-inflammatory cytokines at transcriptional level. Ctrl and USP14^KD^ THP-1 cells were treated for different time periods with or without LPS and relative mRNA levels of pro-inflammatory cytokines (IL-6, IL-1β, and TNF-α) were then measured using qRT-PCR. Upon LPS stimulation in Ctrl THP-1 cells, mRNA levels of IL-6, IL-1β, and TNF-α were increased significantly (open bars; Figure 4E, IL-6; Figure 4F, IL-1β; Figure 4G, TNF-α) and marked enhancement was also detected in USP14^KD^ THP-1 cells (open bars vs. closed bares; Figure 4E, IL-6; Figure 4F, IL-1β; Figure 4G, TNF-α). To verify the production of pro-inflammatory cytokines, intracellular cytokine assay and ELISA were performed. Levels of IL-1β, TNF-α, and IL-6 were significantly elevated in Ctrl THP-1 cells in the presence of LPS (Figure 5A, IL-1β: 1 ± 0.5 vs. 7 ± 1.5; Figure 5B, TNF-α: 2 ± 0.5 vs. 12 ± 2; Figure 5C, IL-6: 2 ± 0.6 vs. 5 ± 1). In addition, their levels were markedly enhanced in USP14^KD^ THP-1 cells compared to those in Ctrl THP-1 cells (Figures 5A,D, IL-1β: 7 ± 1.5 in Ctrl vs. 22 ± 2.5 in USP14^KD^; Figures 5B,E, TNF-α: 12 ± 2 in Ctrl vs. 84 ± 4.5 in USP14^KD^; Figures 5C,F, IL-6: 5 ± 1 in Ctrl vs. 10 ± 1.5 in USP14^KD^). Moreover, production levels of cytokines IL-1β, TNF-α, and IL-6 were consistently elevated in USP14^KD^ THP-1 cells in the presence of LPS stimulation compared to those in Ctrl THP-1 cells (Ctrl vs. USP14^KD^ in Figures 5G,H,I). These results suggest that USP14 can negatively regulate TLR4-mediated NF-κB activation, leading to the inhibition of cytokine production.

### USP14 Induces Deubiquitination of TAB 2 and Inhibits TLR4-Mediated Signaling

**Figure 6:**
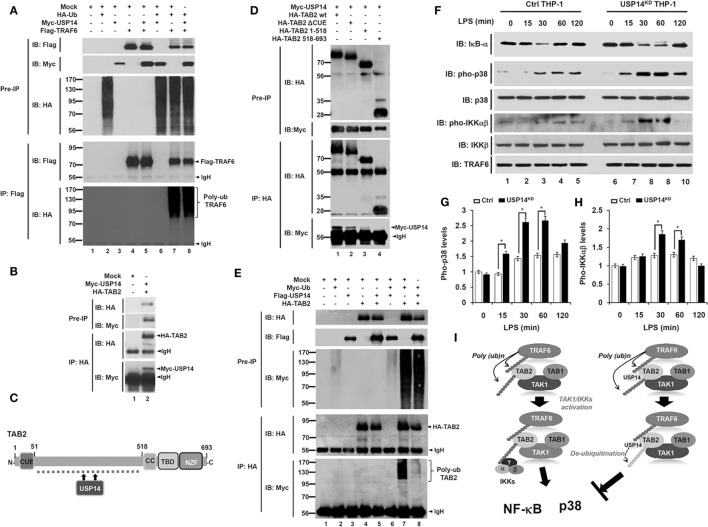
Ubiquitin-specific protease 14 (USP14) induces deubiquitination of TAB 2 and inhibits toll-like receptor 4 (TLR4)-mediated signaling. **(A)** Myc-tagged USP14, HA-tagged Ub, Flag-tagged tumor necrosis factor (TNF) receptor-associated factor 6 (TRAF6), and mock as control plasmid were co-transfected as indicated. At 38 h post-transfection, transfected cells were extracted, immunoprecipitated with anti-Flag antibody, and subjected to IB assay using anti-Flag, anti-Myc, or anti-HA antibody. **(B)** HEK293T cells were transiently transfected with mock as control plasmid, Myc-tagged USP14, or HA-tagged TAB 2. After 38 h, IP assay was performed using anti-HA antibody followed by IB assay using anti-Myc or anti-HA antibody. **(C)** A schematic model showing interactions between TAB 2 and USP14. **(D)** HA-tagged TAB 2 wild type (wt), HA-tagged TAB 2 truncated mutants, and mock as control plasmid were co-transfected with Myc-tagged USP14 into HEK293T cells followed by immunoprecipitation and western blotting analysis. **(E)** HEK293T cells were co-transfected with mock as control plasmid, Myc-tagged Ub, Flag-tagged USP14, or HA-tagged TAB 2 as indicated. At 38 h post-transfection, cells were extracted and immunoprecipitated with anti-HA antibody followed by an IB assay using anti-Myc, anti-Flag, or anti-HA antibody. **(F)** Ctrl THP-1 and USP14^KD^ THP-1 cells were treated with or without LPS (200 ng/ml) for different time periods and then western blot assay was performed using anti-IκB-α, anti-pho-p38, anti-p38, anti-pho-IKKαβ, anti-IKKβ, and anti-TRAF6 antibody. **(G,H)** Band intensity of pho-p38 **(G)** and pho-IKKαβ **(H)** were analyzed with Image J (bottom). Data shown are averages from a minimum of three independent experiments. *P < 0.05. **(I)** A schematic model showing how USP14 regulates TLR4 signaling. Following TLR4 stimulation, ubiquitinated TRAF6 is associated with the TAB 2–TAK1–TAB 1 complex through interaction between its polyubiquitinated chain and TAB 2. TAB 2 is then ubiquitinated and the complex then facilitates the activation of TAK1. Simultaneously, the IKK complex is associated with the former complex through polyubiquitinated chain and TAB 2, leading to the activation of nuclear factor-kappa B (NF-κB) and p38 (left). In contrast, interaction between TAB 2 and USP14 may lead to deubiquitination of TAB 2, which inhibits the association of IKKs, thereby inhibiting the activation of NF-κB and p38 (right).

Based on results shown above, we sought to determine the molecular mechanism by which USP14 negatively regulated TLR4-mediated signaling. In TLR-mediated signaling, TRAF6 acts as an E3 ubiquitin ligase associated with dimeric ubiquitin-conjugating enzyme Ubc13/Uev1A. It then functions as both an adaptor and an E3 ubiquitin ligase by conjugating K63-linked ubiquitin chain to other proteins and activating NF-κB (21, 41). Since USP14 interacted with the CC domain of TRAF6 (Figure 1E), we examined whether USP14 induced deubiquitination of TRAF6. HEK293T cells were transiently transfected with Myc-USP14, Flag-TRAF6, or HA-Ub vectors, and an IP assay was performed using anti-Flag antibody. Polyubiquitination of TRAF6 was significantly induced in the presence of HA-Ub, with ubiquitination level significantly higher than that in the absence of HA-Ub (Figure 6A, lane 4 vs. lane 7). However, no significant change in TRAF6 ubiquitination could be seen in the presence of Myc-USP14 (Figure 6A, lane 7 vs. lane 8), indicating that USP14 was not involved in the deubiquitination of TRAF6.

Although the precise molecular mechanism by which TAB 2 is involved in toll/IL-1 signaling remains unclear, it has been reported that ubiquitination of TAB 2 induced by TRAF6 activates NF-κB and production of pro-inflammatory cytokines through toll/IL-1 signaling complex containing TRAF6, TAK1, and IκB kinase (24). We, therefore, examined whether USP14 was implicated in the deubiquitination of TAB 2. HA-TAB 2 protein was specifically precipitated with Myc-USP14 protein (Figure 6B, lane 2), indicating that USP14 interacted with TAB 2. TAB 2 consists of two different functional domains, CUE and ZnF (Figure 6C), which are important for NF-κB activation (42). We, therefore, tried to identify the interaction site between USP14 and TAB 2. For that purpose, we generated three truncated mutants of TAB 2 (HA-TAB 2 ΔCUE, HA-TAB 2 1-518, and HA-TAB 2 518-693) as described in Section “Materials and Methods.” HEK293T cells were transiently transfected with Myc-USP14, HA-TAB 2 wild type (wt), HA-TAB 2 ΔCUE, HA-TAB 2 1-518, and HA-TAB 2 518-693 vectors and an IP assay was then performed using anti-HA antibody. Myc-USP14 proteins were significantly co-precipitated with HA-TAB 2 wt, HA-TAB 2 ΔCUE, and HA-TAB 2 1-518 (Figure 6D, lanes 1–3), whereas no significant interaction between Myc-USP14 and HA-TAB 2 518-693 was detected (Figure 6D, lane 4). These results suggest that USP14 interacts with the internal domain of TAB 2 as depicted in Figure 6C. We then examined whether USP14 affected the deubiquitination of TAB 2. HEK293T cells were transiently transfected with Flag-USP14, HA-TAB 2, or Myc-Ub vectors as indicated in Figure 6E and an IP assay was performed with anti-HA antibody. The ubiquitination of TAB 2 was significantly induced in the absence of Flag-USP14. However, it was significantly attenuated in the presence of Flag-USP14 (Figure 6E, lane 7 vs. lane 8). These results suggest that USP14 induces the deubiquitination of TAB 2.

Since USP14 induced the deubiquitination of TAB 2, we then examined the functional regulation of TLR4-mediated signaling in Ctrl and USP14^KD^ THP-1 cells. Ctrl and USP14^KD^ THP-1 cells were treated with or without LPS for different time periods and the activation of TLR4 downstream signaling molecules was analyzed by western blotting. Upon LPS stimulation, degradation of IκB-α was significantly increased and delayed in USP14^KD^ THP-1 cells compared to that in Ctrl THP-1 cells (Figure 6F, IB: IκB-α). In the presence of LPS stimulation, phosphorylation of p38 and IKKαβ was significantly increased in Ctrl THP-1 cells in a time-dependent manner compared to that in cells without LPS stimulation (Figure 6F, IB: pho-p38 and IB: pho-IKKαβ). Interestingly, levels of pho-p38 and pho-IKKαβ were markedly higher in USP14^KD^ THP-1 cells than those in Ctrl THP-1 cells (Ctrl THP-1 vs. USP14^KD^ THP-1 cell in Figure 6F, IB: pho-p38 and IB: pho-IKKαβ). Increases of pho-p38 and pho-IKKαβ in USP14^KD^ THP-1 cells were significant (open bars in Ctrl vs. closed bars in USP14^KD^ THP-1 cells in Figures 6G,H). Taken together, these results suggest that TRAF6 and TAB 2 proteins are ubiquitinated upon TLR4 stimulation, which induce the assembly of a signaling complex containing TRAF6, TAK1, and IκB kinase, leading to activation of NF-κB and p38 (Figure 6I, left). In contrast, the interaction between USP14 and TAB 2 induces deubiquitination of TAB 2, resulting in the inhibition of activation of NF-κB and p38 (Figure 6I, right).

## Discussion

In this study, we demonstrated that USP14 and Beclin 1 competitively interacted with the CC domain of TRAF6. This inhibited Beclin 1 ubiquitination, leading to inhibition of autophagy induction. Furthermore, we demonstrated that USP14 induced deubiquitination of TAB 2, which could be ubiquitinated by TRAF6. This suppressed the activation of TLR4-mediated signaling molecules such as TAK1 and IKKs, leading to inhibition of NF-κB activation induced by TLR4 stimulation. Our findings provide a novel molecular mechanism in which USP14 regulates autophagy induction and NF-κB activation upon TLR4 stimulation.

**Figure 7:**
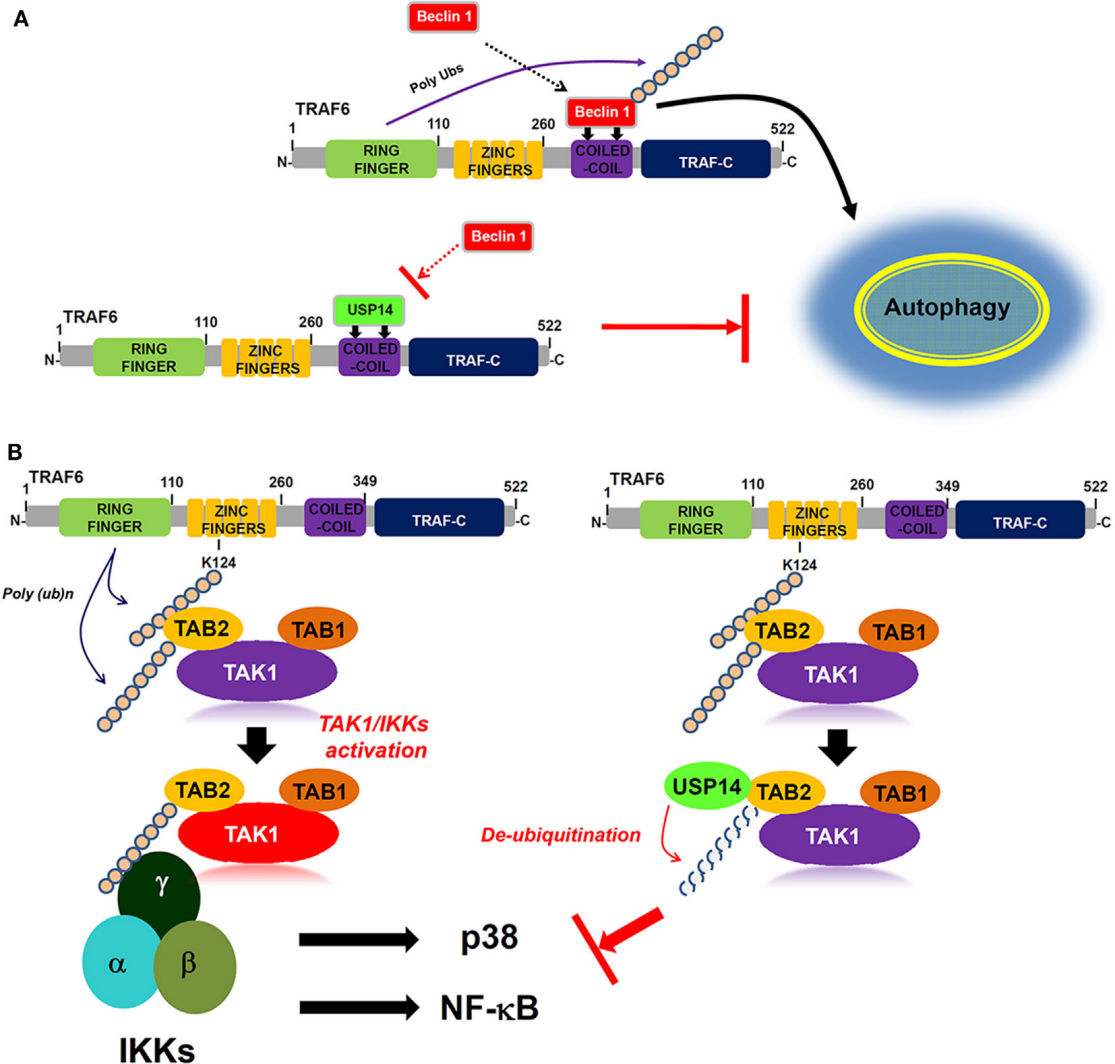
Ubiquitin-specific protease 14 (USP14) negatively regulates autophagy induction and toll-like receptor 4 (TLR4)-mediated signaling. **(A)** Upon TLR4 stimulation, tumor necrosis factor (TNF) receptor-associated factor 6 (TRAF6) interacts with Beclin 1 through its coiled coil (CC) domain, induces ubiquitination of Beclin 1, and facilitate autophagy induction (upper). In contrast, USP14 competitively interacts with Beclin 1 to the CC domain of TRAF6. This leads to the suppression of Beclin 1 ubiquitination by TRAF6, resulting in the inhibition of autophagy induction (down). **(B)** Following TLR4 stimulation, ubiquitinated TRAF6 is associated with the TAB 2–TAK1–TAB 1 complex through an interaction between its polyubiquitinated chain and TAB 2. TAB 2 is then ubiquitinated and the complex facilitates the activation of TAK1. Simultaneously, the IKK complex is associated with the former complex through the polyubiquitinated chain and TAB 2, leading to the activations of nuclear factor-kappa B (NF-κB) and p38 (left). In contrast, the interaction between TAB 2 and USP14 may lead to the deubiquitination of TAB 2, thus inhibiting the association of IKKs, which inhibits the activation of NF-κB and p38 (right).

Ubiquitin-specific protease-14 as a deubiquitinating enzyme plays a key role in intracellular degradative process by trimming K48 ubiquitin chains on proteasome-bound substrates and also involved in K63 deubiquitination process (2, 43, 44). Akt-mediated phosphorylation of USP14 at Ser432 activates its deubiquitinating activity *in vitro* and in cells (30). Additionally, it has been reported that inhibition of USP14 *in vivo* leads to the increased levels of K63-linked ubiquitin conjugates in both spinal cords and neurons (40), suggesting that its K63 deubiquitinating activity is likely to be physiologically relevant. A recent report has shown that USP14 regulates autophagy by suppressing K63 ubiquitination of Beclin 1 (13). Akt-regulated USP14 activity can modulate both proteasomal degradation and autophagy through controlling K48 and K63 ubiquitination, respectively (13). Nevertheless, the precise molecular mechanism by which USP14 is implicated in autophagy induction by inhibiting Beclin 1 ubiquitination remains unclear. We found that USP14 interacted with the CC domain of TRAF6. Since TRAF6 ubiquitin-ligase activity is critical for autophagy induction through Beclin 1 ubiquitination (30), USP14 might be implicated in autophagy induction through regulating ubiquitin-ligase activity of TRAF6. However, USP14 and TRAF6 interaction was not affected by TRAF6 ubiquitin-ligase activity or TRAF6 autoubiquitination. Interestingly, biochemical studies in the present study revealed that USP14 and Beclin 1 competitively interacted with the CC domain of TRAF6. This inhibited TRAF6-mediated ubiquitination of Beclin 1. Although the role of USP14 cannot be completely ruled out as a proteasome-associated DUB enzyme that can affect the expression of TRAF6 and its associated proteins, the inhibitory effect of USP14 on the Beclin 1 ubiquitination might be thought to be based on the inhibition of the molecular interaction between TRAF6 and Beclin 1 proteins rather than by its proteasome-associated DUB enzyme. Functionally, reduction of USP14 in USP14^KD^ THP-1 cells resulted in enhanced autophagy induction upon TLR4 stimulation. Moreover, USP14^KD^ cancer cells USP14^KD^ MDA-MB-231 and USP14^KD^ SK-HEP-1 showed increased ability of migration and invasion. Our results demonstrated that USP14 could inhibit TRAF6-mediated ubiquitination of Beclin 1 by interrupting the molecular interaction between TRAF6 and Beclin 1 as depicted in Figure 7A.

Another regulatory role of USP14 revealed in this study is that USP14 negatively regulates TLR4-mediated signaling for the activation of NF-κB. Ubiquitination and deubiquitination mechanisms are emerging as important regulators of innate and adaptive immune cell signaling (23). TRAF6, the most commonly known ubiquitin ligase in TLR- and IL-1R-mediated signaling, is associated with the dimeric ubiquitin-conjugating enzyme Ubc13/Uev1A. It functions as both an adaptor and an E3 ubiquitin ligase by conjugating K63-linked ubiquitin chain to other proteins (21–23, 41). TRAF6 ubiquitination involves the activation of ubiquitin-dependent kinase TAK1 followed by the binding to TAK1 to several different proteins such as TAB 1, TAB 2, TAB 3, and TAB 4 (20–22). TAB 2 is ubiquitinated by TRAF6, which facilitates the assembly of a toll/IL-1 signaling complex containing TRAF6, TAK1, and IκB kinase (24). A recent report has shown that TRAF2/6-mediated NLRC5 ubiquitination frees IKKα and IKKβ, resulting in the formation of the activated IKK complex containing IKKα, IKKβ, and IKKγ, which activates NF-κB (45). On the other hand, USP14 induced the deubiquitination NLRC5, enhancing NLRC5-mediated inhibition of IKK-NF-κB signaling (45). These results suggest that USP14 specifically enhanced NLRC5-mediated inhibition of NF-κB activation through the inhibition of NLRC5 ubiquitination *via* its DUB activity. Although the molecular mechanism is a little different with the previous report, we demonstrate another regulatory role of USP14 in TLR4-mediated NF-κB activation. We found that USP14 interacted with TRAF6. It is conceivable that USP14 may be involved in TRAF6-mediated ubiquitination of TLR-mediated signaling molecules. Although USP14 and TRAF6 interaction did not affect TRAF6 ubiquitination, we found that USP14 interacted with TAB 2 and induced deubiquitination of TAB 2. We found that overexpression of USP14 in 293/TLR4 cells suppressed NF-κB activity through TLR4 stimulation. Conversely, USP14^KD^ THP-1 cells clearly enhanced NF-κB activation and the production of proinflammatory cytokines such as TNF-α, IL-6, and IL-1β. These results demonstrate that USP14 and TAB 2 interaction can induce deubiquitination of TAB 2 that is ubiquitinated by TRAF6, leading to inhibition of NF-κB activation induced by TLR4 as depicted in Figure 7B.

Ubiquitination and deubiquitination mechanisms are recently emerging as important regulators for many cellular processes, including cellular signals and autophagy induction. In this regard, our results contribute to the understanding of the role of ubiquitination and deubiquitination in regulation of TLR-mediated signaling as well as autophagy induction.

## Author Contributions

Conception/design of study: EC and K-YL. Acquisition of data: YM, M-JK, and SL. Analysis/interpretation of data: YM, M-JK, EC, and K-YL. Drafting the manuscript: EC and K-YL. Important intellectual content: YM, SL, M-JK, EC, and K-YL. Approval of manuscript: MY, SL, M-JK, EC, and K-YL.

## Conflict of Interest Statement

The authors declare that the research was conducted in the absence of any commercial or financial relationships that could be construed as a potential conflict of interest.

## Supplementary Material

Figure S1 USP14 interacts with Beclin 1. **(A)** Expression vectors of Myc-tagged Beclin 1 and Flag-tagged USP14 were co-transfected into HEK293T cells. At 38-h post-transfection, transfected cells were extracted, immunoprecipitated with anti-Myc antibody, and subjected to IB assay using anti-Flag or anti-Myc antibody. **(B)** A schematic model showing the interaction of USP14 to Beclin 1.

Figure S2 Generation of USP14-knockdown (USP14^KD^) THP-1 cells. THP-1 cells were infected with lentivirus containing shRNA targeted human USP14 or control lentivirus according to the manufacture’s protocol. Control THP-1 (Ctrl) and USP14-knockdown THP-1 (USP14^KD^ THP-1) were cultured in puromycin-containing medium (4 μg/ml) for 2 weeks to select stable clones, and immunoblotting with antibody to anti-USP14 or anti-GAPDH was performed to evaluate the knockdown efficacy.

Figure S3 A schematic model showing the inhibition of autophagy formation by USP14. Beclin 1 interacts with the coiled coil domain of TRAF6, and then beclin 1 is ubiquitinated by TRAF6, involving the formation of autopahgy. In contract, USP14 interrupts the interaction of Beclin 1 to TRAF6 through the competitive interaction to TRAF6, results in the inhibition of Beclin 1 ubiquitination by TRAF6.
