# Supplementary figures and images for: Ubiquitin-Specific Protease 14 Negatively Regulates Toll-Like Receptor 4-Mediated Signaling and Autophagy Induction by Inhibiting Ubiquitination of TAK1-Binding Protein 2 and Beclin 1
Source: Front Immunol. 2017 Dec 15;8:1827. doi: 10.3389/fimmu.2017.01827 (PMC5736539; doi:10.3389/fimmu.2017.01827)

**A**

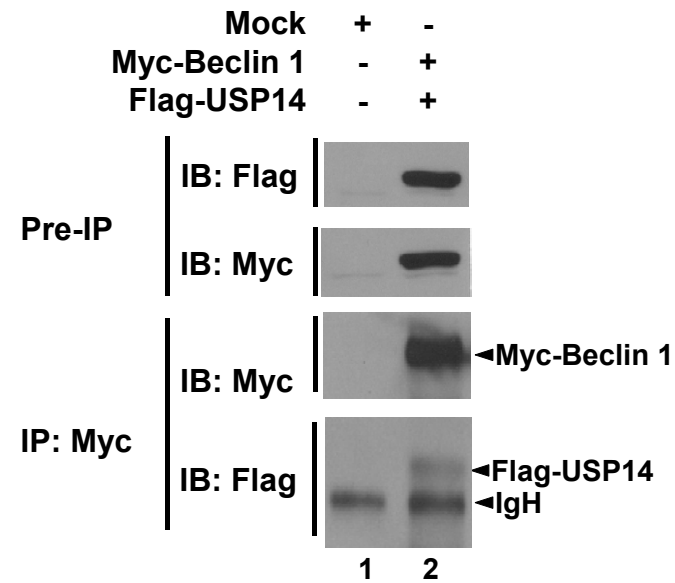

**B**

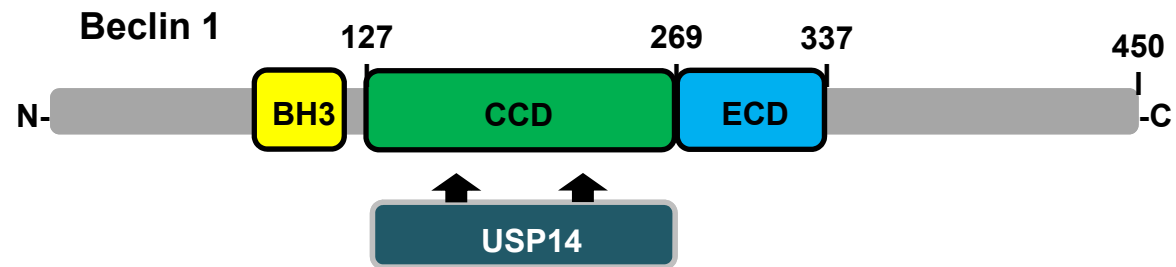

Min et al., Supplementary Figure S1

Supplement: Figure S1 — USP14 interacts with Beclin 1. (A) Expression vectors of Myc-tagged Beclin 1 and Flag-tagged USP14 were co-transfected into HEK293T cells. At 38-h post-transfection, transfected cells were extracted, immunoprecipitated with anti-Myc antibody, and subjected to IB assay using anti-Flag or anti-Myc antibody. (B) A schematic model showing the interaction of USP14 to Beclin 1. [file Image_1.PDF]

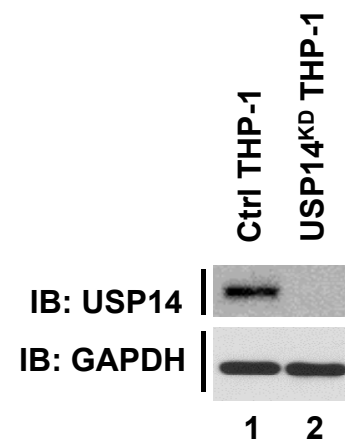

**Min et al., Supplementary Figure S2**

Supplement: Figure S2 — Generation of USP14-knockdown (USP14KD) THP-1 cells. THP-1 cells were infected with lentivirus containing shRNA targeted human USP14 or control lentivirus according to the manufacture’s protocol. Control THP-1 (Ctrl) and USP14-knockdown THP-1 (USP14KD THP-1) were cultured in puromycin-containing medium (4 μg/ml) for 2 weeks to select stable clones, and immunoblotting with antibody to anti-USP14 or anti-GAPDH was performed to evaluate the knockdown efficacy. [file Image_2.PDF]

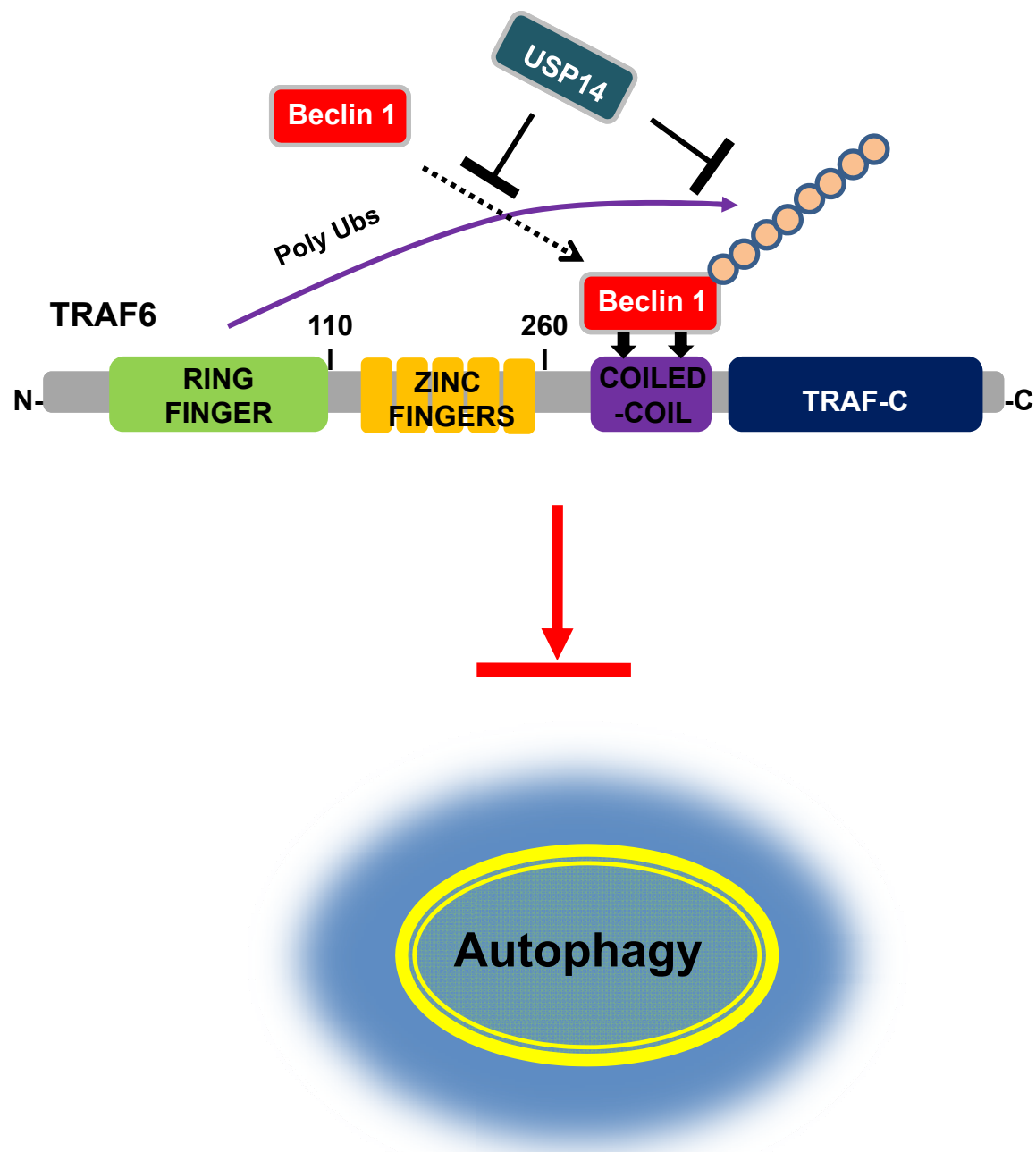

Min et al., Supplementary Figure S3

Supplement: Figure S3 — A schematic model showing the inhibition of autophagy formation by USP14. Beclin 1 interacts with the coiled coil domain of TRAF6, and then beclin 1 is ubiquitinated by TRAF6, involving the formation of autopahgy. In contract, USP14 interrupts the interaction of Beclin 1 to TRAF6 through the competitive interaction to TRAF6, results in the inhibition of Beclin 1 ubiquitination by TRAF6. [file Image_3.PDF]
