# Supplementary material for: Ubiquitin-Specific Protease 14 Negatively Regulates Toll-Like Receptor 4-Mediated Signaling and Autophagy Induction by Inhibiting Ubiquitination of TAK1-Binding Protein 2 and Beclin 1
Source: Front Immunol. 2017 Dec 15;8:1827. doi: 10.3389/fimmu.2017.01827 (PMC5736539; doi:10.3389/fimmu.2017.01827)
Supplement: Supplementary file 4 [file Table_1.docx]

**Table S1.** Primers used for PCR amplification of Beclin 1 truncated mutants

Truncated mutants Direction Primer (5'-3')

Beclin 1 1-127 F TAA GGATCC**ATGGAAGGGTCTAAGACG**

R CGG ATCGAT**TCACGACATGATGTCAAAAAG**

Beclin 1 1-269 F TAA GGATCC**ATGGAAGGGTCTAAGACG**

R GGC ATCGAT **TCAGACGTTGGTTTTCTT**
