# Supplementary material for: Ubiquitin-Specific Protease 14 Negatively Regulates Toll-Like Receptor 4-Mediated Signaling and Autophagy Induction by Inhibiting Ubiquitination of TAK1-Binding Protein 2 and Beclin 1
Source: Front Immunol. 2017 Dec 15;8:1827. doi: 10.3389/fimmu.2017.01827 (PMC5736539; doi:10.3389/fimmu.2017.01827)
Supplement: Supplementary file 5 [file Table_2.docx]

**Table S2.** Primers used for PCR amplification of TAB 2 truncated mutants

Truncated mutants Direction Primer (5'-3')

TAB2 ΔCUE F CGGAATTC ATGAATTTTTCAGATGATTCTGG

R TATGTCGAC TCAGAAATGCCTTGGCATC

TAB2 1-518 F GCGAATTC ATGGCCCAAG

R TATGTCGACTCAACTTATTCTATCCACATGTGCTAA

TAB2 518-693 F ACGAATTC ATGAGTGAAACACGGAAACTGAGTATG

R TATGTCGAC TCAGAAATGCCTTGGCATC
